# Lithium as add-on to quetiapine XR in adult patients with acute mania: a 6-week, multicenter, double-blind, randomized, placebo-controlled study

**DOI:** 10.1186/s40345-014-0014-9

**Published:** 2014-11-08

**Authors:** Michel S Bourin, Emanuel Severus, Juan P Schronen, Peter Gass, Johan Szamosi, Hans Eriksson, Hongally Chandrashekar

**Affiliations:** Department of Pharmacology, Faculty of Medicine, University of Nantes, 98, rue Joseph Blanchart, 44100 Nantes, France; Department of Psychiatry and Psychotherapy, Technical University of Dresden, Dresden, Germany; Cape Trial Centre, Cape Town, South Africa; Department of Psychiatry and Psychotherapy, Central Institute of Mental Health, Medical Faculty Mannheim, Heidelberg University, Heidelberg, Germany; AstraZeneca R&D, Södertälje, Sweden; Bangalore Medical College and Research institute, Victoria Hospital, Bangalore, India

**Keywords:** Bipolar mania, Lithium, Quetiapine XR

## Abstract

Quetiapine extended release (XR) and lithium are treatments with proven efficacy in acute mania. This randomized study evaluated the efficacy and safety of lithium or placebo as add-on to quetiapine XR in adult patients with manic or mixed symptoms of bipolar I disorder. In this 6-week, double-blind study (Trial D144AC00003), adult patients with *DSM-IV-TR-*diagnosed bipolar I disorder (current episode manic or mixed), a Young Mania Rating Scale (YMRS) total score ≥20, and score ≥4 on two of four core YMRS items were administered quetiapine XR (400 to 800 mg/day) and randomly assigned to receive add-on lithium (600 to 1,800 mg/day) or placebo. The primary efficacy end point was change in the YMRS total score from baseline to day 43, analyzed using a mixed-model for repeated measures (MMRM) approach. Secondary efficacy and safety end points were also measured. Rating scales were administered by trained staff. Three hundred fifty-six patients treated with quetiapine XR were randomized to add-on lithium (*n* = 173) or placebo (*n* = 183). Two hundred ninety-one patients (81.7%) completed the study. At day 43, least squares mean change in YMRS total score was −22.8 for add-on lithium and −20.1 for add-on placebo, a statistically significant treatment group difference of −2.69 (*p* < 0.001). On secondary measures, add-on lithium was associated with significant improvements in response, remission, illness severity, and overall illness versus add-on placebo (*p* < 0.05). The number needed to treat was 9.1 for response and 7.9 for remission for add-on lithium compared with add-on placebo. Lithium in combination with quetiapine XR was generally well tolerated, with a similar profile to quetiapine XR in combination with placebo. The addition of lithium to quetiapine XR therapy was associated with significantly greater efficacy than placebo as add-on and was generally well tolerated in patients with acute bipolar I mania. This study was registered under Clinicaltrials.gov Identifier NCT00931723.

**Electronic supplementary material:**

The online version of this article (doi:10.1186/s40345-014-0014-9) contains supplementary material, which is available to authorized users.

## Background

Bipolar disorder is a complex, debilitating illness that typically follows a chronic and recurrent course. Manic, depressed, or mixed symptoms range in severity and rate of onset and in their most extreme forms require hospitalization (Sanchez-Moreno et al. 2009).

Given the severe impact and rapid onset of manic symptoms in many patients, prompt and effective control of symptoms is a primary treatment goal (Garlow 2008; Oral 2005). Guidelines typically recommend lithium, divalproex, or an atypical antipsychotic in the treatment of acute mania, frequently as monotherapy initially, with combination therapy in cases of inadequate response (Connolly and Thase 2011; Goodwin 2009; Grunze et al. 2009; Nivoli et al. 2011; Yatham et al. 2009). Guidelines also recommend combination therapy as first-line treatment for severe manic symptoms (Goodwin 2009; Grunze et al. 2009; Nivoli et al. 2011). However, there is a lack of evidence to support the efficacy and safety of many combination therapies, and not all agents demonstrating antimanic efficacy as monotherapy offer additional efficacy and acceptable tolerability when used in combination (Brooks et al. 2011; Geoffroy et al. 2012; Sachs and Gardner-Schuster 2007; Smith et al. 2007).

Quetiapine extended release (quetiapine XR) demonstrates efficacy as monotherapy in acute mania (Cutler et al. 2011) and bipolar depression (Suppes et al. 2010). Yatham et al. (2004) previously reported that quetiapine immediate release (IR) added to lithium or divalproex provided superior efficacy in treating mania when compared with lithium or divalproex alone. The potential benefits of adding lithium to quetiapine XR have not previously been investigated. The current study compared the efficacy and safety of lithium versus placebo as add-on to quetiapine XR in adults with bipolar I disorder with a current or recent episode of severe manic or mixed symptoms.

## Methods

### Study design

This was a 6-week, multicenter, double-blind, randomized, parallel-group placebo-controlled study (Trial D144AC00003; Clinicaltrials.gov ID: NCT00931723), conducted at 38 study centers in eight countries (Belgium, Bulgaria, Germany, India, Poland, Russia, South Africa, and Ukraine) between June 24, 2009 and November 22, 2010. The study included an enrollment period (with medication washout for 7 to 28 days, according to medication) and a 6-week treatment period, when quetiapine XR was administered as flexible dose to all patients. Patients were randomly assigned in a 1:1 ratio to receive, in addition, either flexible-dose lithium or placebo at the start of treatment. Following mandatory hospitalization during randomization, patients were permitted to continue study treatment as outpatients.

The study was performed in agreement with the ethical principles of the Declaration of Helsinki and was consistent with the International Conference on Harmonization (ICH)/Good Clinical Practice (GCP). Written informed consent was provided by all patients.

### Patient population

Males or females aged 18 to 65 years with a *Diagnostic and Statistical Manual for Metal Disorders, Fourth Edition, Text Revision* (*DSM-IV-TR)* diagnosis of bipolar I disorder (most recent episode manic or mixed) (American Psychiatric Association 2000), confirmed by the Structured Clinical Interview for *DSM-IV* (First et al. 2007), were eligible for inclusion. Patients were required to have a Young Mania Rating Scale (YMRS) (Young et al. 1978) total score ≥20 and ≥4 on two of four core items (irritability, speech, content, disruptive/aggressive behavior), a Clinical Global Impressions for Bipolar Disorder (CGI-BP) (Spearing et al. 1997) score ≥4 (i.e., moderately ill), and to have experienced ≥1 manic or mixed episode (other than the current episode) in the past 5 years.

Key exclusion criteria included current *DSM-IV-TR* Axis I disorders other than bipolar mania; >8 mood episodes within the previous 12 months; mania-like syndrome associated with a medical condition or treatment, substance abuse, or withdrawal; continuous hospitalization for an acute episode of bipolar mania for >3 weeks before randomization; current substance dependence or abuse; current serious suicidal or homicidal risk; or history of nonresponse to study treatments.

The use of an antipsychotic other than quetiapine, mood stabilizer, antidepressant, anxiolytic, hypnotic, other psychoactive drug, or inducer/inhibitor of cytochrome P 3A4 enzymes was prohibited within 7 to 28 days before randomization. Nonpsychoactive and anticholinergic medications were allowed during the study, as were zolpidem tartrate (maximum 10 mg/day), zaleplon (20 mg/day), zopiclone (7.5 mg/day), chloral hydrate (1 g/day), and lorazepam (2 mg/day).

### Study treatments

Open-label quetiapine XR was administered once-daily in the evening, at a starting dose of 300 mg/day on day 1, increasing to 600 mg/day on day 2, and adjusted between 400 and 800 mg/day from day 3 onward depending on efficacy and tolerability, in agreement with prescribing guidelines (Seroquel XR (quetiapine fumarate) 2013). For patients who received quetiapine prior to the study, the existing dose was administered on day 1 and increased to 600 mg at day 2 if the previous dose was lower.

Patients were treated with lithium or placebo twice daily. Lithium (immediate release) was administered at a starting dose of 600 mg on days 1 and 2, increasing to 900 mg/day on days 3 to 8. Lithium (or placebo) dosing was adjusted (range, 600 to 1,800 mg/day) from day 9 at the discretion of the investigator to minimize side effects or achieve target trough concentrations of 0.8 to 1.2 mEq/L. On days 8, 15, 22, 29, and 43, lithium was administered after serum lithium sampling. On each occasion that a lithium dose recommendation was sent for a patient randomized to lithium, a matching sham recommendation was sent to non-lithium patients in relation to the dose of placebo capsule.

The lithium and placebo capsules were identical in appearance, smell, and taste to maintain blinding. In addition, blood samples were drawn from all patients (whether treated with lithium or placebo) on days 8, 15, 22, 29, and 43 for determination of trough serum lithium concentrations.

### Efficacy assessments

The primary outcome measure was change in YMRS total score from baseline to day 43. Secondary outcome measures included changes to day 43 in scores for individual YMRS items, Clinical Global Impressions for Bipolar Disorder-Severity of Illness (CGI-BP-S), CGI-BP-Change (CGI-BP-C) (Spearing et al. 1997), Positive and Negative Syndrome Scale (PANSS) total and activation and positive subscales (Kay et al. 1987), and Montgomery-Åsberg Depression Rating Scale (MADRS) (Montgomery and Asberg 1979). Response (i.e., ≥50% reduction in YMRS total score to day 43), remission (YMRS total score ≤12 at day 43), and improvement in overall bipolar illness (CGI-BP-C score of ‘much’ or ‘very much’ improved overall bipolar illness at day 43) were also assessed, using conventional cutoff criteria (Bourin and Thibaut 2013). Rating scales were administered by staff who were trained in their use and who were blinded to study treatments.

### Safety and tolerability assessments

Safety and tolerability assessments included treatment-emergent adverse events (TEAEs; incidence and severity), adverse event (AE)-related withdrawals, and changes from baseline in vital signs, laboratory parameters, and body weight. Extrapyramidal symptoms (EPS) were assessed by TEAEs, changes in EPS rating scales (Simpson-Angus Scale [SAS] (Simpson and Angus 1970), Abnormal Involuntary Movement Scale [AIMS] (Guy 1976), and Barnes Akathisia Rating Scale (BARS) (Barnes 1989)), worsening of SAS, AIMS, or BARS score category, and initiation of anticholinergic medication for EPS. Suicidality was assessed by TEAEs and prospective use of the Columbia-Suicide Severity Rating Scale (C-SSRS), with mapping of C-SSRS responses also to the Columbia-Classification Algorithm for Suicide Assessment (C-CASA) (Posner et al. 2007, 2011). Other TEAEs of special interest, besides EPS and suicidality, included somnolence, diabetes mellitus, QTc prolongation, neutropenia/agranulocytosis, and depression/depressed mood.

### Statistical analysis

It was estimated that 166 evaluable patients were required in each treatment group to reject the null hypothesis of no difference with a power of 80% when using a two-sided *t*-test at an overall type I error rate of 5%, assuming a true effect of four points on YMRS between the two groups and a standard deviation (SD) of 9.

Efficacy analyses were performed on the modified intent-to-treat analysis set (ITT), which included all randomized patients who received ≥1 dose of quetiapine XR and lithium or placebo and had a baseline YMRS score and ≥1 post-baseline score assessment. Group differences in change in efficacy rating scale score were analyzed using a mixed-model for repeated measures (MMRM) approach; while group differences in response, remission, and overall bipolar illness were tested using generalized estimating equations. The robustness of the primary study analysis was tested using the per-protocol (PP) set (i.e., all ITT patients without major protocol violations or deviations affecting efficacy) and in the ITT set using robust variance estimates, as well as by analysis of covariance (ANCOVA) modeling using last observation carried forward (LOCF) methodology, including baseline YMRS total score as covariate, treatment group as fixed effect, and centers as random effect.

Safety variables were evaluated on the safety analysis set (all randomized patients who received at least one dose of study medication) and are presented using descriptive statistics.

All statistical comparisons were based on a two-sided test using a significance level of 5%. Analyses were performed with SAS® software, Version 9.2 or higher (SAS Institute, Cary, NC, USA).

## Results

**Figure 1:**
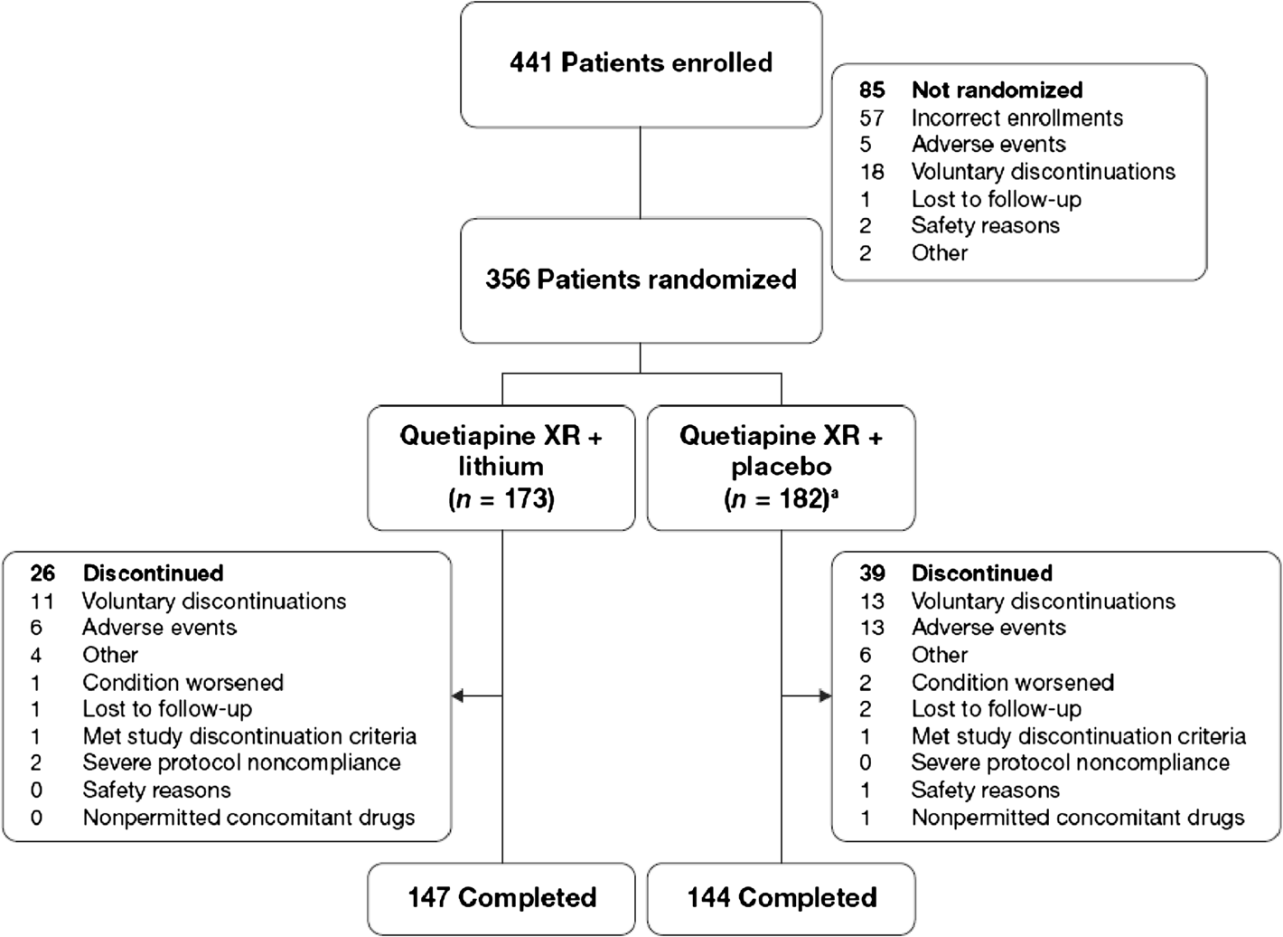
**Patient disposition.** ^**a**^ **One randomized patient did not receive study treatment.**

Overall, 441 patients were enrolled and screened, of whom 356 received quetiapine XR and were randomized to add-on treatment with lithium (*n* = 173) or placebo (*n* = 182) (Figure 1). The most common reason for nonrandomization was incorrect enrollment due to patients not meeting the inclusion criteria (*n* = 57). The majority of patients (91.0%) received study treatment as outpatients. Forty-four of the patients (12.4%) were diagnosed with severe mania and psychotic features at baseline.

The ITT and safety analysis sets comprised 349 and 356 patients, respectively. The PP set included 143 patients (*n* = 45, add-on lithium; *n* = 98, add-on placebo). Failure to achieve lithium concentrations within the target range (*n* = 112 patients) was the most common protocol deviation. Baseline demographics and disease characteristics were generally similar between the treatment groups (Table 1). Mean YMRS total scores at baseline were 29.9 and 30.0 in the add-on lithium and add-on placebo groups, respectively, indicating a severely ill population.

**Table 1:** **Baseline demographic and clinical characteristics (safety set)**

**Demographic and clinical characteristics**	**Quetiapine XR + Lithium (** ***n*** **= 173)**	**Quetiapine XR + Placebo (** ***n*** **= 183)**	**Total (** ***n*** **= 356)**
Age (years)			
Mean (SD)	37.9 (12.7)	38.8 (12.1)	38.3 (12.4)
Median	37.0	38.0	37.0
Range (min, max)	18, 64	18, 65	18, 65
Gender, *n* (%)			
Male	101 (58.4)	121 (66.1)	222 (62.4)
Female	72 (41.6)	62 (33.9)	134 (37.6)
Race, *n* (%)			
White	98 (56.6)	102 (55.7)	200 (56.2)
Asian	73 (42.2)	78 (42.6)	151 (42.4)
Other	2 (1.2)	3 (1.6)	5 (1.4)
Weight (kg)			
Mean (SD)	69.1 (15.9)	71.0 (17.1)	70.1 (16.5)
Median	68.0	69.0	68.0
Range (min, max)	37, 120	37, 115	37, 120
BMI (kg/m^2^)			
Mean (SD)	24.3 (4.3)	24.9 (5.2)	24.6 (4.8)
Median	23.8	24.1	23.9
Range (min, max)	15.8, 37.1	14.7, 43.8	14.7, 43.8
Psychiatric diagnosis, *n* (%)			
Most recent episode manic	159 (91.8)	173 (94.5)	332 (93.3)
Most recent episode mixed	14 (8.1)	10 (5.5)	24 (6.7)
Rapid cycling, *n* (%)			
Yes	3 (1.7)	5 (2.7)	8 (2.2)
No	170 (98.3)	178 (97.3)	348 (97.8)
Time since first diagnosis of acute mania, years			
Mean (SD)	7.1 (6.6)	7.8 (7.0)	7.5 (6.8)
Time since first mania/mixed episode, years			
Mean (SD)	7.2 (6.6)	8.1 (7.0)	7.7 (6.8)
Total number of mania/mixed episodes in the past year, *n* (%)			
0	56 (32.4)	56 (30.6)	112 (31.5)
1	88 (50.9)	88 (48.1)	176 (49.4)
≥ 2	29 (16.8)	39 (21.3)	68 (19.1)
Previous medication use, *n* (%)			
Lithium	4 (2.3)	2 (1.1)	6 (1.7)
Quetiapine	8 (4.6)	17 (9.3)	25 (7.0)

Open-label quetiapine XR was administered at a mean modal dose of 623.1 mg/day (range, 200 to 800 mg/day) in the add-on lithium group and 669.9 mg/day (range, 300 to 900 mg/day) in the add-on placebo group. The mean modal lithium dose was 1,085.5 mg/day (range, 300 to 1,800 mg/day). The mean serum lithium level was 0.72 mEq/L (range, 0.00 to 1.43 mEq/L) at day 43; mean lithium levels did not differ notably during the study (range of means, 0.64 to 0.77 mEq/L). Mean durations of exposure to quetiapine XR were 38.5 days (range, 2 to 48) in the add-on lithium group and 36.9 days (range, 1 to 48) in the add-on placebo group; mean durations of exposure to add-on lithium or placebo were 38.6 days (range, 3 to 48) and 37.2 days (range, 2 to 48), respectively.

The use of concomitant medications was similar between the lithium (24.9%) and placebo (23.0%) add-on groups, with anilides (acetaminophen) representing the most common concomitant medication in both groups (>3% each) (Additional file 1: Table S1).

A total of 291 (81.7%) patients completed the study, while 65 (18.3%) patients discontinued the study prematurely (*n* = 26 (15.0%), add-on lithium; *n* = 39 (21.3%), add-on placebo group), most commonly because of voluntary discontinuation (Figure 1). The majority of patients were compliant with quetiapine XR (98.5%, add-on lithium; 94.4%, add-on placebo group) and with lithium (99.2%) or placebo (96.1%).

### Efficacy assessments

#### Primary efficacy end point

**Figure 2:**
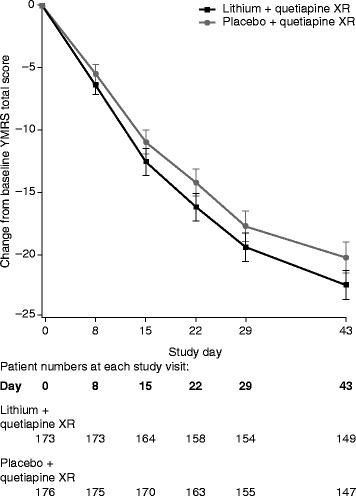
**Mean change in YMRS total score (with 95% CIs) from baseline (observed case data, ITT set).**

Mean YMRS total scores decreased from baseline to day 43 in both treatment groups (Figure 2). Least squares (LS) mean (SE) reductions in YMRS total score were −22.8 (0.71) in the add-on lithium and −20.1 (0.71) in the add-on placebo group, a significant between-group difference of −2.69 (95% CI, −4.09 to −1.29; *p* < 0.001) (Table 2).

**Table 2:** **Mean changes from baseline to day 43 in primary and secondary efficacy measures (ITT set)**

**Measure**	**Quetiapine XR + Lithium (** ***n*** **= 173)**	**Quetiapine XR + Placebo (** ***n*** **= 176)**
**Young Mania Rating Scale (YMRS) total score**		
LS Mean change (SE) (MMRM)	−22.8 (0.71)	−20.1 (0.71)
Mean difference (95% CI)	−2.69 (−4.09, −1.29)	
*p*-value	**<0.001**	
Mean change (SD) (OC)	−22.6 (7.24)	−20.4 (7.64)
*n* at day 43	149	147
**Clinical Global Impressions Bipolar–Severity (CGI-BP-S)**		
LS Mean change (SE) (MMRM)	−2.5 (0.08)	−2.2 (0.08)
Mean difference (95% CI)	−0.24 (−0.43, −0.04)	
*p*-value	**0.017**	
Mean change (SD) (OC)	−2.5 (0.99)	−2.3 (1.00)
*n* at day 43	149	147
**Clinical Global Impressions Bipolar-Change (CGI-BP-C)**		
LS Mean (SE) (MMRM)	1.7 (0.09)	1.9 (0.09)
Mean difference (95% CI)	−0.20 (−0.37, −0.03)	
*p*-value	**0.020**	
Mean (SD) (OC)	1.7 (0.69)	1.9 (0.85)
*n* at day 43	149	147
**Positive and Negative Syndrome Scale (PANSS) total score**		
LS Mean change (SE) (MMRM)	−19.2 (0.91)	−15.6 (0.91)
Mean difference (95% CI)	−3.7 (−5.74, −1.63)	
*p*-value	**<0.001**	
Mean change (SD) (OC)	−19.8 (12.34)	−16.1 (10.82)
n at Day 43	148	147
**PANSS positive subscale**		
LS Mean change (SE) (MMRM)	−8.1 (0.32)	−7.0 (0.32)
Mean difference (95% CI)	−1.1 (−1.85, −0.39)	
*p*-value	**0.003**	
Mean change (SD) (OC)	−8.1 (4.47)	−7.1 (4.54)
*n* at day 43	148	147
**PANSS activation subscale**		
LS Mean change (SE) (MMRM)	−7.1 (0.28)	−5.9 (0.28)
Mean difference (95% CI)	−1.2 (−1.77, −0.61)	
*p*-value	**<0.001**	
Mean change (SD) (OC)	−7.3 (3.60)	−5.9 (3.77)
*n* at day 43	148	147
**Montgomery-Åsberg Depression Rating Scale (MADRS) total score**		
LS Mean change (SE) (MMRM)	−4.8 (0.37)	−4.1 (0.37)
Mean difference (95% CI)	−0.6 (−1.36, 0.07)	
*p*-value	**0.077**	
Mean change (SD) (OC)	−5.0 (4.84)	−4.5 (3.56)
*n* at day 43	149	147

ITT, intent to treat; LS, least squares; MMRM, mixed-model repeated measures; OC, observed cases. A negative change in score indicates improvement, with the exception of Clinical Global Impressions Bipolar-Change, where a positive change in score indicates improvement. Significant *p*-values shown in bold.

The robustness of the primary efficacy analysis was confirmed by supportive analyses, where significant between-group differences at day 43 were observed in MMRM analysis of the PP set (*p* = 0.005), MMRM modeling of the ITT set with robust variance estimates (*p* < 0.001), and ANCOVA modeling with LOCF methodology (*p* = 0.001).

In *post hoc* analysis of patients categorized by serum lithium level, LS mean (SE) change in YMRS total score was −23.6 (0.79) in the ≥6 mEq/L and −21.6 (1.04) in the <6 mEq/L group (*p* < 0.001 and 0.171, respectively, vs. placebo).

#### Secondary efficacy end points

**Figure 3:**
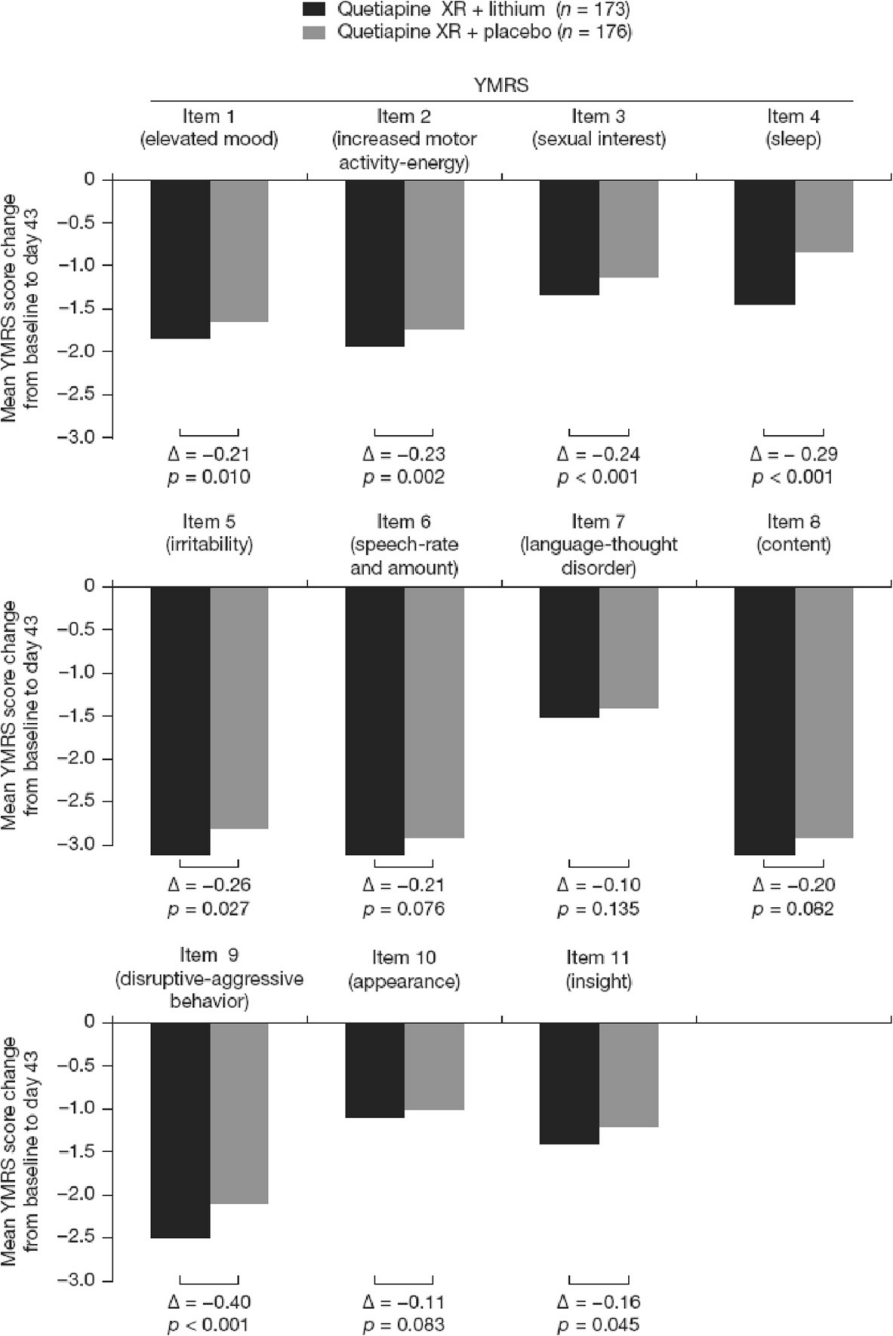
**Mean changes in YMRS item scores from baseline (ITT set, MMRM analysis).**

Response (≥50% reduction in YMRS score at day 43) occurred in 79.2% and 68.2% of the lithium and placebo add-on groups, respectively, and remission (YMRS total score ≤12 at day 43) was reported in 72.3% and 59.7%, respectively. Between-group differences in response and remission rates were significant in favor of add-on lithium (*p* = 0.005, both). The number needed to treat was 9.1 for response and 7.9 for remission for add-on lithium compared with add-on placebo. The majority of individual YMRS item scores were also significantly improved in the lithium versus the placebo add-on group (Figure 3).

In *post hoc* analyses of patients categorized by serum lithium level, response rates were 85.1% and 72.7%, respectively, in the ≥6 and <6 mEq/L groups (*p* = 0.006 and 0.164, respectively, vs. placebo). Remission rates in the respective lithium groups were 78.1% and 65.5% (*p* = 0.004 and 0.170, respectively, vs. placebo).

Add-on lithium was associated with significantly greater LS mean reductions in CGI-BP-S (mean difference, −0.24, *p* = 0.017) and CGI-BP-C (mean difference, −0.20; *p* =0.020) than add-on placebo. Proportions of patients ‘much improved’ or ‘very much improved’ at day 43 were similar in the two groups (77.5% vs. 71.0%; *p* = 0.177).

Psychotic features and agitation and aggression, assessed by PANSS total and positive and activation subscales, were improved in both treatment groups, with significantly greater improvements in the add-on lithium than add-on placebo group (*p* < 0.001, 0.003, and < 0.001, for respective scales; Table 2). Severity of depressive symptoms, measured by MADRS total score, improved in both groups, without significant between-group difference (Table 2).

### Safety and tolerability assessments

The incidence of TEAEs was 63.0% in the add-on lithium and 48.1% in the add-on placebo group. Tremor, somnolence, dizziness, diarrhea, and vomiting occurred at higher rates in the add-on lithium than add-on placebo group (Table 3). Treatment-emergent adverse events in both groups were mostly mild to moderate in intensity.

**Table 3:** **Incidence of treatment-emergent adverse events (≥2% in any group; safety set)**

**Adverse event,** ***n*** **(%)**	**Quetiapine XR + Lithium (** ***n*** **= 173)**	**Quetiapine XR + Placebo (** ***n*** **= 183)**
Tremor	27 (15.6)	9 (4.9)
Somnolence	22 (12.7)	10 (5.5)
Constipation	16 (9.2)	16 (8.7)
Dry mouth	14 (8.1)	14 (7.7)
Dizziness	11 (6.4)	8 (4.4)
Insomnia	11 (6.4)	12 (6.6)
Headache	9 (5.2)	11 (6.0)
Pyrexia	10 (5.8)	9 (4.9)
Diarrhea	8 (4.6)	2 (1.1)
Vomiting	8 (4.6)	0
Sedation	5 (2.9)	3 (1.6)
Dysarthria	4 (2.3)	2 (1.1)
Nausea	5 (2.9)	3 (1.6)
Weight increased	2 (1.2)	5 (2.7)
Increased appetite	4 (2.3)	1 (0.5)

Patients with multiple events falling under the same category are counted only once in that category.

Adverse events led to discontinuation in a greater proportion of patients in the add-on placebo than add-on lithium group (*n* = 13 (7.1%) and *n* = 6 (3.5%), respectively). Psychiatric disorders, including mania, were the most common AE leading to discontinuation, reported in two patients in the add-on lithium and seven patients in the placebo add-on group. Serious adverse events (SAEs) included mania (*n* = 3 patients) and gastroenteritis (*n* = 1) in the add-on lithium group and mania (*n* = 6), aggression (*n* = 1), hostility (*n* = 1), and no therapeutic response (*n* = 1) in the add-on placebo group. The SAEs of emergent mania led to study discontinuation in two patients in the add-on lithium and six patients in the add-on placebo group.

Incidences of TEAEs potentially related to EPS were 16.8% in the add-on lithium and 6.6% in the add-on placebo group, including tremor in 15.6% and 4.9% of patients, respectively. All these TEAEs were mild to moderate in intensity. Two TEAEs (tremor and dystonia) resulted in study discontinuation in the add-on placebo group. The majority of patients showed either improvement or no change in EPS severity during the study, assessed by SAS, AIMS, and BARS scores. For example, SAS score was improved in 11.6% and 8.2% of the add-on lithium and placebo-add groups, respectively, unchanged in 67.1% and 75.4% and worsened in 15.6% and 9.8%, respectively, at day 43. No patients initiated anticholinergic medication for new-onset EPS.

No TEAEs potentially related to suicidality were reported during the study. Columbia-Suicide Severity Rating Scale assessment identified two patients with suicidal behavior and one with suicidal ideation in the add-on lithium group. Columbia-Suicide Severity Rating Scale and C-CASA assessments at end of treatment indicated that no patients had any type of suicidal ideation in either group. Rates of emergent depression were low in both add-on lithium (1.2%) and add-on placebo (0.5%) groups.

Among other TEAEs of special interest, there were no reported events potentially related to diabetes mellitus, neutropenia, or QTc prolongation. No clinically meaningful between-group differences were reported in incidences of physical examination, vital signs, hematology, clinical chemistry, or urinalysis findings (Additional file 1: Table S2). There were no notable between-group differences in mean changes in liver function parameters, electrolytes, or lipids. The mean change in prolactin concentration was greater in the add-on lithium than add-on placebo group (mean (SD) –210.8 (488.1) mU/L vs. −116.9 (602.4) mU/L). A greater proportion of patients in the add-on lithium group had weight gain ≥7% at end of treatment compared with add-on placebo (8.0% vs. 4.7%).

## Discussion

In this 6-week study, lithium as an add-on therapy to quetiapine XR was significantly more effective than add-on placebo for improving acute severe symptoms of bipolar mania, measured by change in YMRS total score at day 43. Reductions in YMRS score of 22.8 and 20.1, respectively, occurred in the add-on lithium and add-on placebo groups, with a clinically relevant between-group difference of 2.69 in favor of add-on lithium (*p* < 0.001). Consistent with this primary efficacy measure, the secondary efficacy measures showed that add-on lithium significantly improved a range of manic symptoms and the overall severity of illness compared with add-on placebo.

The combination of quetiapine XR with lithium was generally well tolerated, and reported AEs were consistent with the known safety profiles of these medications in bipolar disorder (Bowden 2000; Cutler et al. 2011; Suppes et al. 2010). In agreement with studies of quetiapine IR (Emsley et al. 2005), worsening of glycemic control was not observed during quetiapine XR treatment combined with lithium or placebo. Incidences of potentially EPS-related TEAEs were also consistent with studies of quetiapine IR combined with lithium or divalproex (Suppes et al. 2009; Yatham et al. 2004). Tremor, a known side effect of lithium (Bowden 2000), occurred at higher rates in the add-on lithium than add-on placebo group. More patients in the add-on lithium than add-on placebo group also had weight gain ≥7% at the end of treatment. Add-on lithium was not associated with an altered rate of switch to depressive symptoms, which occurred at low incidences in both groups.

The current study is unique in comparing quetiapine XR combined with lithium against quetiapine XR combined with placebo. Direct comparison with previous studies - which compared a mood stabilizer combined with quetiapine IR against a mood stabilizer combined with placebo - is not possible (Sachs et al. 2004; Suppes et al. 2009; Vieta et al. 2008a; Yatham et al. 2004), although all these studies indicate the superior efficacy of combination therapy versus monotherapy. In the earlier studies, quetiapine IR combined with a mood stabilizer was significantly more effective than mood stabilizer alone in acute mania (Sachs et al. 2004; Yatham et al. 2004) and in the prevention of recurrent mood disorder (Suppes et al. 2009; Vieta et al. 2008a). A *post hoc* analysis of the two maintenance studies (Suppes et al. 2009; Vieta et al. 2008a) demonstrated that quetiapine IR combined with lithium was associated with a 68% reduction in risk of recurrence of a mood event (manic, depressive, or mixed) compared with placebo and lithium (*p* < 0.001), while quetiapine combined with divalproex reduced recurrence risk by 72% compared with placebo and divalproex (*p* < 0.001) (Suppes et al. 2013). A second *post hoc* analysis of the maintenance studies showed that quetiapine combined with mood stabilizer (vs. mood stabilizer alone) significantly reduced the risk of recurrence of a mood event, whether the index episode was manic, depressive, or mixed (Vieta et al. 2012) (*p* ≤ 0.001, all). Quetiapine (IR or XR) in combination with lithium or divalproex is also associated with quality-adjusted life expectancy and cost-effectiveness benefits relative to maintenance treatment with mood stabilizers alone (Plosker 2012). Together, these findings may provide a rationale for initiating treatment with combined quetiapine XR and lithium in patients with severe acute manic symptoms, rather than escalating the dose of monotherapy up to the highest dose approved, and hence also for subsequent use of this combination as maintenance treatment to prevent recurrence.

Notable features of this study include the initiation of medications (quetiapine XR and lithium or placebo) concurrently at the start of the study, which reflects management of severe acute mania in practice. To demonstrate efficacy for combination therapy in this setting is challenging, when compared with study of patients who show partial response to monotherapy. This study also required an inpatient status at randomization, which provides reassurance on the reliability of YMRS and other scoring at baseline.

Direct comparison of the quetiapine XR and lithium combination versus other combination therapies in acute mania is hampered by the limited availability of other studies and differences in study design, duration, and patient population. Most studies report superior efficacy for combinations versus monotherapy (measured as symptom control, speed of onset, or relapse prevention), but also increased rates of AEs and related discontinuations (Geoffroy et al. 2012). For example, when compared with a mood stabilizer alone, combinations including a mood stabilizer and olanzapine or asenapine are associated with weight gain, combinations including aripiprazole lead to greater risk of akathisia, and combinations including quetiapine are associated with increased somnolence (Geoffroy et al. 2012; Sachs et al. 2004; Szegedi et al. 2012; Tohen et al. 2002; Vieta et al. 2008b; Yatham et al. 2004). While the quetiapine XR and lithium combination in the current study was associated with elevated rates of tremor and somnolence when compared with quetiapine alone, the combination was generally well tolerated and rates of treatment-related discontinuations were below add-on placebo-group levels.

This study raises a number of questions that would require further investigation. Add-on lithium showed significant benefits despite a high proportion of patients (approximately 65%) having serum lithium levels outside the target range. The target lithium range selected (0.8 to 1.2 mEq/L) was based on evidence for the efficacy of lithium as monotherapy in bipolar disorder (Goodwin and Jamison 2007). *Post hoc* analyses in the current study reported greater efficacy (assessed as YMRS score change, response rate, and remission rate) for add-on lithium versus add-on placebo at a lithium level ≥6 mEq/L, but not <6 mEq/L. These data are consistent with an earlier study that reported significant efficacy against manic symptoms for lithium monotherapy versus placebo at higher (0.72 and 0.5 mEq/kg/day) but not lower (0.24 mEq/kg/day) lithium doses (Stokes et al. 1976). It may also be conjectured that the use of lower quetiapine doses in the add-on lithium than add-on placebo group (mean modal 623.1 mg/day vs. 669.9 mg/day) contributed to reduce the between-group difference in YMRS score. Investigators may have judged that there was no requirement to increase the quetiapine dose in a substantial proportion of patients due to the effective control of manic symptoms achieved by the addition of blinded lithium. Furthermore, the lower doses of quetiapine used in the add-on lithium group may have contributed to the beneficial side effect profile of combination therapy observed in this study.

The study design excluded patients with a known lack of response to lithium or quetiapine, although only 1.7% and 7.0%, respectively, of the study population were treated with lithium and quetiapine previously. The benefits of combination therapy in lithium nonresponders are therefore unknown from this study, although other studies (Hardoy et al. 2005; Yatham et al. 2004) have suggested that lithium nonresponders may benefit from combination therapy with quetiapine and lithium. The pharmacological rationale for combining quetiapine and lithium also remains to be confirmed, although it may be conjectured that the mechanisms of action of quetiapine (Nemeroff et al. 2002; Nord et al. 2011) and lithium (Quiroz et al. 2010; Shim et al. 2012) are complementary and additive as combination therapy.

## Conclusions

Add-on lithium enhanced the efficacy of quetiapine XR in patients with acute bipolar I mania, without compromising safety and tolerability.

## Additional file

Additional file 1: Tables S1 to S2.
**Table S1.** Use of concomitant medications (safety set). **Table S2.** Incidence (*n* (%)) of potentially clinically significant shifts in vital signs and laboratory parameters from normal at baseline to day 43 (safety set).

## Abbreviations

AE: adverse event
AIMS: abnormal involuntary movement scale
ANCOVA: analysis of covariance
BARS: Barnes Akathisia Rating Scale
C-CASA: Columbia-Classification Algorithm for Suicide Assessment
CGI-BP: Clinical Global Impressions for Bipolar Disorder
CGI-BP-C: CGI-BP-Change
CGI-BP-S: CGI-BP-Severity of Illness
C-SSRS: Columbia-Suicide Severity Rating Scale
*DSM-IV-TR*: *Diagnostic and Statistical Manual for Mental Disorders, Fourth Edition, Text Revision*
EPS: extrapyramidal symptoms
IR: immediate release
ITT: intent-to-treat
LOCF: last-observation-carried-forward
LS: least squares
MMRM: mixed-model for repeated measures
PANSS: positive and negative syndrome scale
PP: per protocol
TEAE: treatment-emergent adverse events
XR: extended release
YMRS: Young Mania Rating Scale

## References

[CR1] American Psychiatric Association (2000). Diagnostic and Statistical Manual of Mental Disorders, Fourth Edition, Text Revision.

[CR2] Barnes TR (1989). A rating scale for drug-induced akathisia. Br J Psychiatry.

[CR3] Bourin M, Thibaut F (2013). How to assess drugs in the treatment of acute bipolar mania?. Front Pharmacol (Online ahead of print).

[CR4] Bowden CL (2000). Efficacy of lithium in mania and maintenance therapy of bipolar disorder. J Clin Psychiatry.

[CR5] Brooks JO, Goldberg JF, Ketter TA, Miklowitz DJ, Calabrese JR, Bowden CL, Thase ME (2011). Safety and tolerability associated with second-generation antipsychotic polytherapy in bipolar disorder: findings from the Systematic Treatment Enhancement Program for Bipolar Disorder. J Clin Psychiatry.

[CR6] Connolly KR, Thase ME (2011) The clinical management of bipolar disorder: a review of evidence-based guidelines. Prim Care Companion CNS Disord 13(4)ᅟ. 10.4088/PCC.10r01097 PMC3219517 22132354

[CR7] Cutler AJ, Datto C, Nordenhem A, Minkwitz M, Acevedo L, Darko D (2011). Extended-release quetiapine as monotherapy for the treatment of adults with acute mania: a randomized, double-blind, 3-week trial. Clin Ther.

[CR8] Emsley R, Turner HJ, Schronen J, Botha K, Smit R, Oosthuizen PP (2005). Effects of quetiapine and haloperidol on body mass index and glycaemic control: a long-term, randomized, controlled trial. Int J Neuropsychopharmacol.

[CR9] First MB, Williams JBW, Spitzer RL, Gibbon M (2007). Structured Clinical Interview for DSM-IV-TR Axis I Disorders, Clinical Trials Version (SCID-CT).

[CR10] Garlow SJ (2008). Interventions for acute mood episodes in patients with bipolar disorder. J Clin Psychiatry.

[CR11] Geoffroy PA, Etain B, Henry C, Bellivier F (2012). Combination therapy for manic phases: a critical review of a common practice. CNS Neurosci Ther.

[CR12] Goodwin GM (2009). Evidence-based guidelines for treating bipolar disorder: revised second edition–recommendations from the British Association for Psychopharmacology. J Psychopharmacol.

[CR13] Goodwin FK, Jamison KR (2007). Manic-Depressive Illness: Bipolar Disorders and Recurrent Depression.

[CR14] Grunze H, Vieta E, Goodwin GM, Bowden C, Licht RW, Moller HJ, Kasper S (2009). The World Federation of Societies of Biological Psychiatry (WFSBP) guidelines for the biological treatment of bipolar disorders: update 2009 on the treatment of acute mania. World J Biol Psychiatry.

[CR15] Guy W (1976). ECDEU Assessment Manual for Psychopharmacology. Revised Edition.

[CR16] Hardoy MC, Garofalo A, Carpiniello B, Calabrese JR, Carta MG (2005). Combination quetiapine therapy in the long-term treatment of patients with bipolar I disorder. Clin Pract Epidemiol Ment Health.

[CR17] Kay SR, Fiszbein A, Opler LA (1987). The positive and negative syndrome scale (PANSS) for schizophrenia. Schizophr Bull.

[CR18] Montgomery SA, Asberg M (1979). A new depression scale designed to be sensitive to change. Br J Psychiatry.

[CR19] Nemeroff CB, Kinkead B, Goldstein J (2002). Quetiapine: preclinical studies, pharmacokinetics, drug interactions, and dosing. J Clin Psychiatry.

[CR20] Nivoli AM, Colom F, Murru A, Pacchiarotti I, Castro-Loli P, Gonzalez-Pinto A, Fountoulakis KN, Vieta E (2011). New treatment guidelines for acute bipolar depression: a systematic review. J Affect Disord.

[CR21] Nord M, Nyberg S, Brogren J, Jucaite A, Halldin C, Farde L (2011). Comparison of D(2) dopamine receptor occupancy after oral administration of quetiapine fumarate immediate-release and extended-release formulations in healthy subjects. Int J Neuropsychopharmacol.

[CR22] Oral TE (2005). Treatment of acute mania. Neuro Endocrinol Lett.

[CR23] Plosker GL (2012). Quetiapine: a pharmacoeconomic review of its use in bipolar disorder. Pharmacoeconomics.

[CR24] Posner K, Oquendo MA, Gould M, Stanley B, Davies M (2007). Columbia Classification Algorithm of Suicide Assessment (C-CASA): classification of suicidal events in the FDA's pediatric suicidal risk analysis of antidepressants. Am J Psychiatry.

[CR25] Posner K, Brown GK, Stanley B, Brent DA, Yershova KV, Oquendo MA, Currier GW, Melvin GA, Greenhill L, Shen S, Mann JJ (2011). The Columbia-Suicide Severity Rating Scale: initial validity and internal consistency findings from three multisite studies with adolescents and adults. Am J Psychiatry.

[CR26] Quiroz JA, Machado-Vieira R, Zarate CA, Manji HK (2010). Novel insights into lithium's mechanism of action: neurotrophic and neuroprotective effects. Neuropsychobiology.

[CR27] Sachs GS, Gardner-Schuster EE (2007). Adjunctive treatment of acute mania: a clinical overview. Acta Psychiatr Scand Suppl.

[CR28] Sachs G, Chengappa KN, Suppes T, Mullen JA, Brecher M, Devine NA, Sweitzer DE (2004). Quetiapine with lithium or divalproex for the treatment of bipolar mania: a randomized, double-blind, placebo-controlled study. Bipolar Disord.

[CR29] Sanchez-Moreno J, Martinez-Aran A, Tabares-Seisdedos R, Torrent C, Vieta E, Ayuso-Mateos JL (2009). Functioning and disability in bipolar disorder: an extensive review. Psychother Psychosom.

[CR30] Seroquel XR (quetiapine fumarate) [prescribing information]. Wilmington, DE: AstraZeneca Pharmaceuticals LP. Available at: http://www1.astrazeneca-us.com/pi/seroquelxr.pdf; 2013.

[CR31] Shim SS, Hammonds MD, Tatsuoka C, Feng IJ (2012). Effects of 4-weeks of treatment with lithium and olanzapine on long-term potentiation in hippocampal area CA1. Neurosci Lett.

[CR32] Simpson GM, Angus JW (1970). A rating scale for extrapyramidal side effects. Acta Psychiatr Scand Suppl.

[CR33] Smith LA, Cornelius V, Warnock A, Tacchi MJ, Taylor D (2007). Pharmacological interventions for acute bipolar mania: a systematic review of randomized placebo-controlled trials. Bipolar Disord.

[CR34] Spearing MK, Post RM, Leverich GS, Brandt D, Nolen W (1997). Modification of the Clinical Global Impressions (CGI) Scale for use in bipolar illness (BP): the CGI-BP. Psychiatry Res.

[CR35] Stokes PE, Kocsis JH, Arcuni OJ (1976). Relationship of lithium chloride dose to treatment response in acute mania. Arch Gen Psychiatry.

[CR36] Suppes T, Vieta E, Liu S, Brecher M, Paulsson B (2009). Maintenance treatment for patients with bipolar I disorder: results from a North American study of quetiapine in combination with lithium or divalproex (trial 127). Am J Psychiatry.

[CR37] Suppes T, Datto C, Minkwitz M, Nordenhem A, Walker C, Darko D (2010). Effectiveness of the extended release formulation of quetiapine as monotherapy for the treatment of acute bipolar depression. J Affect Disord.

[CR38] Suppes T, Vieta E, Gustafsson U, Ekholm B (2013). Maintenance treatment with quetiapine when combined with either lithium or divalproex in bipolar I disorder: analysis of two large randomized, placebo-controlled trials. Depress Anxiety.

[CR39] Szegedi A, Calabrese JR, Stet L, Mackle M, Zhao J, Panagides J (2012). Asenapine as adjunctive treatment for acute mania associated with bipolar disorder: results of a 12-week core study and 40-week extension. J Clin Psychopharmacol.

[CR40] Tohen M, Chengappa KN, Suppes T, Zarate CA, Calabrese JR, Bowden CL, Sachs GS, Kupfer DJ, Baker RW, Risser RC, Keeter EL, Feldman PD, Tollefson GD, Breier A (2002). Efficacy of olanzapine in combination with valproate or lithium in the treatment of mania in patients partially nonresponsive to valproate or lithium monotherapy. Arch Gen Psychiatry.

[CR41] Vieta E, Suppes T, Eggens I, Persson I, Paulsson B, Brecher M (2008). Efficacy and safety of quetiapine in combination with lithium or divalproex for maintenance of patients with bipolar I disorder (international trial 126). J Affect Disord.

[CR42] Vieta E, T’joen C, McQuade RD, Carson WH, Marcus RN, Sanchez R, Owen R, Nameche L (2008). Efficacy of adjunctive aripiprazole to either valproate or lithium in bipolar mania patients partially nonresponsive to valproate/lithium monotherapy: a placebo-controlled study. Am J Psychiatry.

[CR43] Vieta E, Suppes T, Ekholm B, Udd M, Gustafsson U (2012). Long-term efficacy of quetiapine in combination with lithium or divalproex on mixed symptoms in bipolar I disorder. J Affect Disord.

[CR44] Yatham LN, Paulsson B, Mullen J, Vagero AM (2004). Quetiapine versus placebo in combination with lithium or divalproex for the treatment of bipolar mania. J Clin Psychopharmacol.

[CR45] Yatham LN, Kennedy SH, Schaffer A, Parikh SV, Beaulieu S, O’Donovan C, MacQueen G, McIntyre RS, Sharma V, Ravindran A, Young LT, Young AH, Alda M, Milev R, Vieta E, Calabrese JR, Berk M, Ha K, Kapczinski F (2009). Canadian Network for Mood and Anxiety Treatments (CANMAT) and International Society for Bipolar Disorders (ISBD) collaborative update of CANMAT guidelines for the management of patients with bipolar disorder: update 2009. Bipolar Disord.

[CR46] Young RC, Biggs JT, Ziegler VE, Meyer DA (1978). A rating scale for mania: reliability, validity and sensitivity. Br J Psychiatry.

