# Supplementary material for: Lithium as add-on to quetiapine XR in adult patients with acute mania: a 6-week, multicenter, double-blind, randomized, placebo-controlled study
Source: Int J Bipolar Disord. 2014 Nov 8;2:14. doi: 10.1186/s40345-014-0014-9 (PMC4224669; doi:10.1186/s40345-014-0014-9)
Supplement: Additional file 1: Tables S1 to S2. — Table S1. Use of concomitant medications (safety set). Table S2. Incidence (n (%)) of potentially clinically significant shifts in vital signs and laboratory parameters from normal at baseline to day 43 (safety set). [file 40345_2014_14_MOESM1_ESM.doc]

**Additional file 1**

**Table S1 Use of concomitant medications (safety set)**

| **Drug class (*n*, %)** | **Quetiapine XR**  **+**  **Lithium**  **(*n* = 173)** | **Quetiapine XR**  **+**  **Placebo**  **(*n* = 183)** |
| --- | --- | --- |
| Number of patients who took other concomitant medication  ACE inhibitors and diuretics  ACE inhibitors, plain  Acetic acid derivatives, related substances  Alpha- and beta-blocking agents  Angiotensin II antagonists and Ca channel blockers  Angiotensin II antagonists, plain  Anilides  Antacids with antiflatulents  Antidiarrheal microorganisms  Antipropulsives  Benzodiazepine derivatives  Benzodiazepine-related drugs  Beta-blocking agents, non-selective  Beta-blocking agents, selective  Biguanides  Bulk producers  Combinations Al, Ca, Mg compounds  Combinations oral blood glucose lowering drugs  Combinations of vitamins  Contact laxatives  Corticosteroids, very potent (group iv)  Dihydropyridine derivatives  Fibrates  Fluoroquinolones  Folic acid and derivatives  H_2_-receptor antagonists  Imidazole derivatives  Imidazoline receptor agonists  Insulins and analogs for injection, intermediate-acting  Iron in other combinations  Leukotriene receptor antagonists  Macrolides  Mucolytics  Multivitamins, plain  Osmotically acting laxatives  Papaverine and derivatives  Penicillins with extended spectrum  Phenylalkylamine derivatives  Piperazine and derivatives  Piperazine derivatives  Propionic acid derivatives  Propulsives  Proton pump inhibitors  Purine derivatives  Renin inhibitors  Selective beta-2-adrenoreceptor agonists  Serotonin (5-HT_3_) antagonists  Substituted alkylamines  Sulfonamides, plain  Sulfonamides, urea derivatives  Sulfur-containing imidazole derivatives  Tertiary amines  Thyroid hormones  Vitamin B1, plain | 43 (24.9)  0  1 (0.6)  1 (0.6)  1 (0.6)  0  1 (0.6)  6 (3.5%)  0  2 (1.2)  0  0  5 (2.9)  3 (1.7)  3 (1.7)  1 (0.6)  2 (1.2)  1 (0.6)  0  0  4 (2.3)  0  1 (0.6)  1 (0.6)  3 (1.7)  1 (0.6)  3 (1.7)  1 (0.6)  1 (0.6)  1 (0.6)  1 (0.6)  1 (0.6)  0  0  0  3 (1.7)  0  0  0  1 (0.6)  2 (1.2)  6 (3.5)  1 (0.6)  3 (1.7)  1 (0.6)  1 (0.6)  1 (0.6)  3 (1.7)  2 (1.2)  0  0  1 (0.6)  2 (1.2)  1 (0.6)  1 (0.6) | 42 (23.0)  1 (0.5)  5 (2.7)  0  0  1 (0.5)  1 (0.5)  6 (3.3)  1 (0.5)  0  1 (0.5)  1 (0.5)  7 (3.8)  0  2 (1.1)  0  5 (2.7)  0  1 (0.5)  1 (0.5)  2 (1.1)  1 (0.5)  3 (1.6)  0  0  0  0  0  1 (0.5)  0  0  0  1 (0.5)  1 (0.5)  2 (1.1)  1 (0.5)  1 (0.5)  1 (0.5)  1 (0.5)  0  2 (1.1)  7 (3.8)  0  2 (1.1)  0  0  0  0  1 (0.5)  1 (0.5)  1 (0.5)  0  1 (0.5)  2 (1.1)  0 |

**Table S2 Incidence (*n* (%)) of potentially clinically significant shifts in vital signs and laboratory parameters from normal at baseline to day 43 (safety set)**

| **Parameter** | **Thresholds for Potentially Significant Values** | **Shifts** | | | |
| --- | --- | --- | --- | --- | --- |
|  |  | **Quetiapine**  **XR + Lithium**  **(*n* = 173)** | | **Placebo**  **(*n* = 183)** | |
|  |  | ***N***^a^ | ***n* (%)** | ***N***^b^ | ***n* (%)** |
| **Vital signs**^b^ |  |  |  |  |  |
| Pulse | ≥ 20 bpm increase | 173 | 1 (0.6) | 177 | 2 (1.1) |
| SBP | ≥ 20 mmHg decrease | 173 | 1 (0.6) | 177 | 0 (0) |
| DBP | ≥ 20 mmHg decrease | 173 | 0 (0) | 177 | 0 (0) |
| **Clinical chemistry** |  |  |  |  |  |
| Alanine aminotransferase (U/L) | ≥ 3 × ULN | 159 | L: NA  H: 0 (0) | 168 | L: NA  H: 0 (0) |
| Aspartate aminotransferase (U/L) | ≥ 3 × ULN | 160 | L: NA  H: 0 (0) | 168 | L: NA  H: 0 (0) |
| Creatinine (µmol/L) | ≥ 120 | 160 | L: NA  H: 0 (0) | 168 | L: NA  H: 0 (0) |
| Glucose, fasting (mmol/L) | ≤ 2.5; ≥ 7.0 | 140 | L: 0 (0)  H: 5 (3.6) | 143 | L: 0 (0)  H: 4 (2.8) |
| HbA_1c_ (%) | > 7.5 | 161 | L: NA  H: 0 (0) | 166 | L: NA  H: 0 (0) |
| Total cholesterol (mmol/L) | ≥ 6.21 | 160 | L: NA  H: 7 (4.4) | 168 | L: NA  H: 13 (7.7) |
| Triglycerides (mmol/L) | ≥ 2.26 | 160 | L: NA  H: 16 (10.0) | 168 | L: NA  H: 30 (17.9) |
| High-density lipoprotein (mmol/L) | ≤ 1.04 | 160 | L: 16 (10.0)  H: NA | 168 | L: 21 (12.5)  H: NA |
| Low-density lipoprotein (mmol/L) | ≥ 4.2 | 156 | L: NA  H: 6 (3.8) | 164 | L: NA  H: 8 (4.9) |
| Thyroid stimulating hormone (mU/L) | > 5 | 158 | L: NA  H: 38 (24.1) | 167 | L: NA  H: 4 (2.4) |
| Free thyroxine (pmol/L) | < 0.8 × LLN; > 1.2 × ULN | 160 | L: 15 (9.4)  H: 1 (0.6) | 167 | L: 15 (9.0)  H: 0 (0) |
| Prolactin (mU/L) | Males (> 645); females (> 968) | 160 | L: NA  H: 2 (1.3) | 167 | L: NA  H: 5 (3.0) |
| **Hematology** |  |  |  |  |  |
| Hematocrit | Males (≤ 0.37; ≥ 0.55);  females (≤ 0.32; ≥ 0.50) | 159 | L: 9 (5.7)  H: 0 (0) | 164 | L: 3 (1.8)  H: 0 (0) |
| Hemoglobin (g/L) | Males (≤ 115; ≥ 185); females (≤ 105; ≥ 165) | 160 | L: 3 (1.9)  H: 0 (0) | 166 | L: 3 (1.8)  H: 1 (0.6) |
| Total red blood cell count (cells/L) | ≤ 3 × 10^12^;  ≥ 6 × 10^12^ | 160 | L: 0 (0)  H: 0 (0) | 166 | L: 0 (0)  H: 1 (0.6) |
| Platelet count (cells/L) | ≤ 100 × 10^9^; ≥ 600 × 10^9^ | 149 | L: 0 (0)  H: 0 (0) | 154 | L: 0 (0)  H: 0 (0) |
| Total white blood cell count (cells/L) | ≤ 3 × 10^9^;  ≥ 16 × 10^9^ | 159 | L: 1 (0.6)  H: 3 (1.9) | 166 | L: 1 (0.6)  H: 1 (0.6) |
| Neutrophils (cells/L) | ≤ 0.5 × 10^9^; ≥ 10.0 × 10^9^ | 158 | L: 0 (0)  H: 4 (2.5) | 164 | L: 0 (0)  H: 3 (1.8) |
| Eosinophils (cells/L) | ≥ 1.0 ×10^9^ | 158 | L: NA  H: 9 (5.7) | 164 | L: NA  H: 4 (2.4) |
| Basophils (cells/L) | ≥ 0.5 × 10^9^ | 158 | L: NA  H: 0 (0) | 164 | L: 0 (0)  H: 0 (0) |
| Lymphocytes  (cells/L) | ≤ 0.5 × 10^9^; ≥ 6.0 × 10^9^ | 158 | L: 1 (0.6)  H: 0 (0) | 164 | L: 0 (0)  H: 0 (0) |
| Monocytes  (cells/L) | ≥ 1.4 × 10^9^ | 158 | L: NA  H: 0 (0) | 164 | L: NA  H: 0 (0) |

^a^*N*, number of patients with baseline and post-baseline assessments.

^b^From supine to standing.

DBP, diastolic blood pressure; H, high; L, low; LLN, lower limit of normal; NA, not applicable (no potentially clinically significant values defined); SBP, systolic blood pressure; ULN, upper limit of normal.

**Additional file The Trial 003 Study Investigators**

Volodymyr Abramov, Regional Clinical Psychiatric Hospital, Donetsk, Ukraine; Valentin Akabaliev, MMA - University Multiprofile Hospital for Active Treatment, Plovdiv, Bulgaria; Aleksander Araszkiewicz, Szpital Uniwersytecki im. Dr A. Jurasza w Bydgoszczy, Bydgoszcz, Poland; Anatoly Bogdanov, Arkhangelsk Regional Clinical Psychiatric Hospital, Arkhangelsk, Russia; Janusz Bukowski, Szpital Specjalistyczny im. H. Klimontowicza w Gorlicach, Gorlice, Poland; Hongally Chandrashekar, Victoria Hospital, Bangalore, India; Mahesh Chudgar, Mental Illness Treatment Rehabilitation Foundation (MITR), Ahmedabad, India; Andre De Nayer, Grand Hopital de Charleroi, Montignies-sur-Sambre, Belgium; Vladislav Demchenko, Kyiv City Psychoneurological Hospital # 2, Kiev, Ukraine; Natalia Dobrovolskaya, SHI City Psychoneurological Dispensary #7, St. Petersburg, Russia; Mahesh Gowda, Spandana Nursing Home, Bangalore, India; Penka Grozeva, State Psychiatric Hospital 'Dr. Georgi Kissiov', Radnevo, Bulgaria; Emil Kaludiev, MMA - University Multiprofile Hospital for Active Treatment, Sofia, Bulgaria; Georgi Krastev, MHAT 'Kazanlak' EOOD, Kazanlak, Bulgaria; Ramesh (Kumar) Mahendru, Mahendru Psychiatric Centre, Kanpur, India; Nataliya Maruta, St. In. "Inst. of Neurol. Psych. and Narcol. of the AMSU", Kharkiv, Ukraine; Svitlana Moroz, Regional Clinical Hospital n.a. 1.1. Mechnicov, Dniproppetrovsk, Ukraine; Umesh Sureshrao Nagapurkar, Sujata Birla Hospital & Research Centre, Nashik, India; Keshava Pai, Kasturba Medical College and Hospital, Mangalore, India; Nadukuru N. Raju, Government Hospital for Mental Care, Visakhapatnam, India; Satheesh Rao, K. S. Hegde Medical Academy, Mangalore, India; Tatyana Runevska, DDPDru Sofia District EOOD, Sofia, Bulgaria; Sofiya Rymsha, Acad. O.I. Yuschenko Regional Psychiatric Hospital M.I. Pirogov VNMU, Vinnitsa, Ukraine; Loris Sayan, DDPDru 'Prof. Dr. Ivan Temkov' EOOD, Bourgas, Bulgaria; Juan Paul Schronen, Cape Trial Centre, Cape Town, South Africa; Emanuel Severus, Ludwig-Maximilians-Universität, München, Germany; Vladislav Shamrey, Military Medical Academy, St. Petersburg, Russia; P.S.V.N. Sharma, Kasturba Hospital, Manipal, India; Oleg Shiryaev, Voronezh State Psychoneurological Dispensary, Voronezh, Russia; Georgiev Svetlozar, State Psychiatry Hospital - 'Pazardzhik', Pazardzhik, Bulgaria; Agata Szulc, Samodzielny Publiczny Psychiatryczny ZOZ, Choroszcz, Poland; Anil Tambi, Dr. Tambi's Neuropsychiatry Centre, Jaipur, India; Vladimir Tochilov, City Psychiatric Hospital #2 of St. Nikolay Chudotvorets, St. Petersburg, Russia; Christo van Dyk, Clinical Project Research, Worcester, South Africa; Vladimir Vilianov, SEIHPE Saratov SMU of Roszdrav b/o Municipal Clinical Hospital, Saratov, Russia; Rakesh Yadav, R.K. Yadav Memorial Mental Health & De-Addiction Hospital, Jaipur, India; Kausar Yakhin, Kazan State Medical University, Kazan, Russia; Gennadiy Zilberblat, MI of KRC Regional Psychiatric & Narcological Medical Union, Glevakha, Ukraine.
